# New App-Based Dietary and Lifestyle Intervention on Weight Loss and Cardiovascular Health

**DOI:** 10.3390/s22030768

**Published:** 2022-01-20

**Authors:** Alejandro Martínez-Rodríguez, María Martínez-Olcina, Juan Mora, Pau Navarro, Nuria Caturla, Jonathan Jones

**Affiliations:** 1 Department of Analytical Chemistry, Nutrition and Food Science, University of Alicante, 03690 Alicante, Spain; maria.martinezolcina@ua.es (M.M.-O.); juan.mora@ua.es (J.M.); 2 Alicante Institute of Health and Biomedical Research (ISABIAL), 03010 Alicante, Spain; 3 Monteloeder S.L., C/Miguel Servet 16, 03203 Elche, Spain; paunavarro@monteloeder.com (P.N.); nuriacaturla@monteloeder.com (N.C.); jonathanjones@monteloeder.com (J.J.)

**Keywords:** personalized nutrition, mhealth, ehealth, mobile app, consumer wearables, dietary intervention, weight management, blood pressure

## Abstract

Consumer digital technology is rapidly evolving, allowing users to manage their health in a simple, non-invasive manner. However, there are few studies revealing if using digital technology as part of an intervention really has an impact in consumer health compared with traditional strategies. The objective of the current study is to compare two groups (MTB; *n* = 18, 46.1 ± 10.4 years and MTBAPP; *n* = 19, 45.3 ± 6.40 years) of overweight, prehypertensive individuals in losing weight and lowering their blood pressure. Both were provided with nutritionist-guided recommendations, a wearable tracking device and a dietary supplement that has previously been proven to help lose body weight and lower blood pressure. In addition, one of the groups (MTBAPP) used a mobile app specifically designed for the intervention. Blood pressure, body composition, triglyceride level, peak expiratory flow, forced expiratory volume in the first second and maximum oxygen volume were measured at different time points. In addition, participants were monitored with an activity bracelet throughout the intervention. As a result, both groups significantly lost body weight, while the group using the app additionally improved blood pressure levels and lowered fat mass. Furthermore, the app users significantly increased the number of daily steps and decreased sedentary time. In conclusion, the addition of a mobile app with daily reminders to follow healthy lifestyle recommendations increased physical activity and overall improved blood pressure and fat mass levels when compared with a group performing the same intervention but in absence of the mobile application.

## 1. Introduction

High blood pressure is one of the most common disorders in the world and is also the main cause for the development of cardiovascular diseases [1]. There are currently 1.13 billion adults worldwide with high blood pressure (>140/90 mmHg). The reason for such a high incidence is generally contributed to excess body weight, lack of physical exercise, unhealthy eating habits, rising prevalence of diabetes [2], and excess consumption of alcohol [3], salt, and tobacco use [4]. One of the major problems with high blood pressure is that in the majority of cases its presence is unknown to the patient, due to the lack of noticeable symptoms.

When an individual is first diagnosed with raised blood pressure or pre-hypertension, the first recommended strategy is to change lifestyle habits, in the form of a healthy diet and physical exercise [5]. In the majority of cases, high blood pressure is accompanied by excess body weight, therefore it is also important to include weight loss in the intervention.

Individuals are increasingly interested in adopting preventive strategies to avoid the onset of chronic disease. Therefore, besides improving their eating habits and increasing physical activity, there is an increased consumption of natural products that can help reduce body weight and blood pressure; thus, hopefully avoiding or delaying pharmacological treatment [6,7,8].

At the same time, the use of digital technology in the form of consumer wearables and mobile apps is on the rise, with one in six individuals worldwide using at least one device [9]. Consumer devices’ technological advances are reaching a point where their use in treatment adherence and follow-ups are becoming popular both for the users as well as health professionals [10]. Considering that around half of the patients taking blood pressure medication are non-compliant [11], it is important to find additional strategies to increase adherence. Thus, the use of mobile apps can help increase compliance.

Whereas wearable devices allow tracking of physical activity, the use of a mobile app can contribute to increases in activity adoption, providing support and motivation [4]. Furthermore, these apps linked to the devices can estimate the energy use of the participant, allowing nutritionists to calculate the energy demands and plan their dietary recommendations accordingly [12].

In this vein, the development of health-related mobile apps for personal use has increased substantially in recent years, with over 325,000 apps in the stores [13]. The most downloaded apps are those related to physical activity and weight management, therefore demonstrating that the users are ready to adopt these tools.

It is hypothesized that the combined use of this dietary supplement, along with the nutritional and lifestyle recommendations, product intake reminders, and behavior tracking provided by the app, as well as the monitoring of activity through the wearable device, will provide a holistic approach to help users manage their health and ultimately lower their blood pressure and lose weight. Therefore, the aim of this study was to evaluate the effectiveness of a mobile application to manage user behavior and potentially improve health outcomes in prehypertensive and overweight individuals.

## 2. Materials and Methods

### 2.1. Study Design

This human intervention study comprised of two groups. Both groups were provided with dietary and activity recommendations, were controlled by a nutritionist monthly, and provided with a wearable device (Fitbit Charge 2) and the dietary supplement Metabolaid^®^. Group 1 (MTB) included all the aforementioned, while group 2 (MTBAPP) also installed in their cell phones an app specifically designed for the study. The subjects were randomized following the recommendations published by Mahmoud Saghaei, 2011 [14].

### 2.2. Intervention

#### 2.2.1. Dietary Supplement Formulation

The dietary supplement used was a combination of purified extracts of lemon verbena leaves (*L. citriodora*) and hibiscus flower (*H. sabdariffa*), called Metabolaid^®^. This ingredient was provided by Monteloeder SL (Alicante, Spain). Details on the composition and characterization of the blend can be found in previous publications [15,16]. The subjects were instructed to take 2 capsules per day, 30 min before breakfast. Each capsule contained 250 mg of Metabolaid^®^ and 120 mg of excipient (cellulose microcrystalline).

#### 2.2.2. Mobile Application

A mobile application was specifically designed for the study. The app design was performed by Monteloeder, while another company, 7sense mHealth Behavioral Solutions SL, performed the development. The app was installed in the cell phones of the users, who were previously informed of the type of data that was to be collected through the app and their data privacy rights and signed a written consent. The user installed the app, registered using their email address, and integrated their Fitbit account with the app. Once they were on the home page, they set an alarm through the app to remind them to take the dietary supplement daily. On the home page they could see and track their progress on blood pressure and body weight, which they input manually. Other parameters that were measured were their water intake (how many glasses of water are taken daily), how many portions of fruits and vegetables, and minutes of physical activity. Water and fruits/vegetable intake was inputted manually by the user, while physical activity was collected through the Fitbit account.

**Figure 1:**
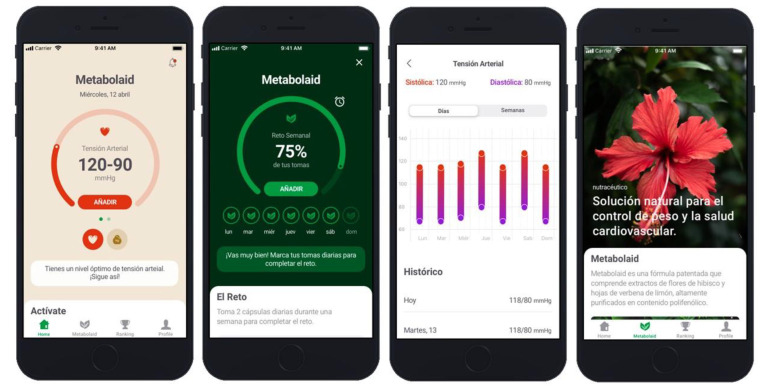
App Images.

Educational material was included in each section (dietary supplement, water intake, fruits/vegetables, and physical activity). The users were recommended to participate in a weekly challenge through the app, to motivate lifestyle changes. The challenges comprised of simple tasks, such as motivating to increase their water or fruit/vegetable intake or do more exercise. The users were recommended to take 8 glasses of water, 5 pieces of fruits/vegetables, and perform 30 min of physical activity per day. In addition, a tip on how to adopt that lifestyle change in their daily life was sent daily. The users were rewarded with points when reaching the daily recommendation and for complying with the weekly challenge. These points were then presented in a chart in the app where all the users were ranked. This was performed in order to incentivize and motivate the users (Figure 1).

### 2.3. Subjects

**Figure 2:**
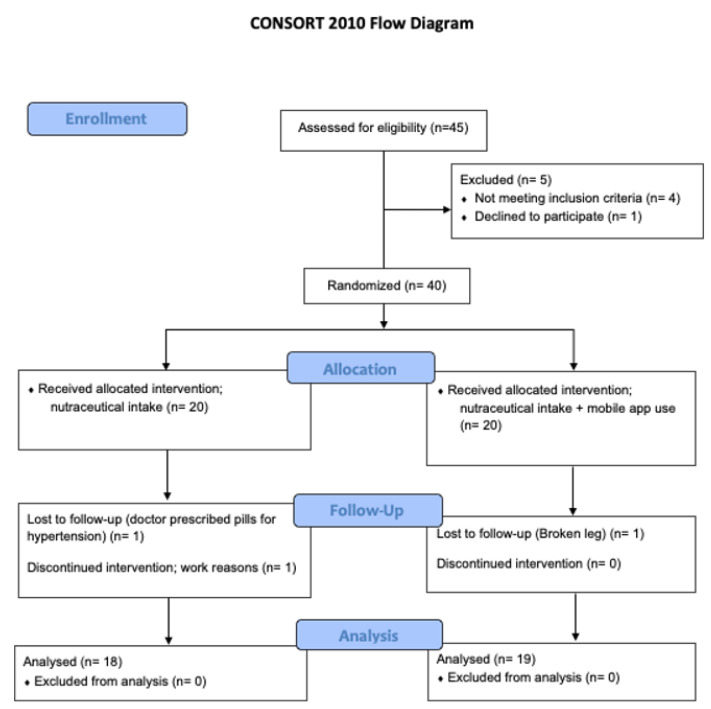
Consort 2010 Flow Diagram.

Subjects were recruited in a private clinic located in the city of Elche (Spain). The study commenced in September 2020 and ended in April 2021. The inclusion criteria were: adults over 18 years of age, BMI > 25 kg/m^2^, blood pressure of 120–140 mmHg/80–90 mmHg for systolic/diastolic measurements, no chronic medication or presence of any chronic disorder, and to also possess a smartphone where the mobile app can be installed. The subjects were informed of the study details and signed a written consent. Exclusion criteria included: the presence of a chronic condition related to obesity, blood pressure, or cholesterol; use of any medication for the before-mentioned disorders; medication/supplement for weight management or cardiovascular health; frequent consumption of alcohol; and in the case of women, being pregnant or lactating. A total of 40 subjects were initially recruited, 37 of which completed the full 3-month study (Figure 2).

### 2.4. Trial Design

The study was divided into various phases: (1) initial evaluation; (2) follow-up at 1 and 2 months; and (3) final evaluation at 3 months. During these phases, the subjects were assessed for body weight, blood pressure, height, body composition through electrical bioimpedance (fat %, water %, fat-free mass, bone density, metabolic age, and visceral fat); anthropometric measurements (waist and hip circumference, abdominal perimeter, triceps fold, biceps fold, subscapular fold, wrist, and femur diameters), lung capacity by spirometry, peak expiratory flow (PEF), triglycerides, and a physical test consisting in walking 1 mile. The participants were always appointed at the same time in all the visits to ensure they were in the same conditions (meals, exercise, rest).

### 2.5. Declarations: Ethical Approval, Consent to Participate and Consent for Publication

The present study was carried out in agreement with the standards of the Helsinki Declaration. The Ethics Committee for Human Research of the University of Alicante (Spain) approved the clinical trial (UA-2020-04-16). The researchers maintained the confidentiality of all personal data. The study was also registered in clinicaltrials.gov (Ref: NCT04828655).

### 2.6. Data Collection

#### 2.6.1. Blood Pressure

A single-arm blood pressure measurement was performed in each visit using the Omron RS2 wrist blood pressure monitor. The participants were seated and asked to relax for 10 min prior to performing the measurement. Both diastolic blood pressure (DBP) and systolic blood pressure (SBP) were measured at each review.

#### 2.6.2. Body Composition—Bioimpedance

Body composition was evaluated by bioelectric impedance with a Tanita BC-730F (Amsterdam, The Netherlands), as it is a non-invasive, low cost, and commonly used approach for BC measurements and assessment of clinical condition [17]. The variables analyzed were weight, BMI, % fat and visceral fat. It has been found that there is sufficient correlation (r-values) between Tanita and the four-compartment model for body fat measurement [18].

#### 2.6.3. Body Composition (BC)—Anthropometric Measurements

Regarding anthropometric measurements, the data obtained from each subject were: weight, height, waist circumference, hip circumference, mean abdominal circumference, subscapular fold, biceps fold, triceps fold, mean abdominal circumference, hip circumference, and waist circumference. The standard protocol of the International Society for the Advancement of Kynanthropometry (ISAK) was followed [19]. Height was measured using a SECA stadiometer (1 mm accuracy). Girths were measured using a Lufkin metric tape (1 mm accuracy), a Smartmet small sliding caliper was used to measure breadths (1 mm accuracy), and for the skinfolds a Harpenden skinfold caliper was used (0.2 mm accuracy).

#### 2.6.4. Triglycerides

Blood samples were extracted and triglycerides measured using an Accutrend^®^ Plus. The Accutrend^®^ Plus test uses capillary serum and is based on the retention of blood cells by filtration through fiberglass when a drop of blood is applied on the reactive strip. An enzymatic reaction on the strip occurs when exposed to oxygen, giving rise to a color. The reflectance of the strip is measured at 660 nm, and triglyceride concentration is calculated using a simple algorithm. The precision of the Accutrend^®^ Plus, as indicated by the product information, is 3.4% [20].

#### 2.6.5. Peak Expiratory Flow (PEF) and Forced Expiratory Volume in the First Second (FEV1)

As previously commented [21], all participants performed a conventional spirometry as well as an additional one with the Air Smart spirometer, following Spanish Society of Pulmonology and Thoracic Surgery (SEPAR) guidelines in order to obtain acceptable and reproducible results [22]. With this method, both PEF (maximum speed achieved when forcing air out of the lungs) and FEV1 (amount of air forced out of the lungs in 1 s) were assessed.

#### 2.6.6. Maximum Oxygen Uptake (VO_2max_)

A Rockport 1 mile effort test was performed [23], which is a standard submaximal effort test. A 3–5 min warm-up was performed before the start of the test, led by a physical activity and sports professional. Basic joint mobility exercises were performed. Treadmill speed was gradually increased until subjects reached their maximum walking speed that they could maintain for 1 mile. Heart rate was monitored throughout the test using a Fitbit watch.

#### 2.6.7. Remote Physical Activity Tracking

Each subject was provided with a Fitbit Charge 2 activity tracker (Fitbit Inc., San Francisco, CA, USA) for the whole duration of the study. The activity tracker allows measurement of heart rate as well as various physical activity parameters: daily steps, distance, calories burned, minutes, and intensity of physical activity, as well as sleep duration and time in different sleep phases [24].

#### 2.6.8. Diet Record

A diet diary (dietary record) was provided during the first and last week of the study, to perform a quantitative analysis of the diets. This method consisted of asking the subjects to write down daily during those days the food and beverages they ingested. The intake of each participant was calculated by averaging the 7 days, using the DietoPro diet program. In addition, in each of the measurements, the participants gave feedback on their sensations.

#### 2.6.9. Subject Opinion on App

In order to determine the participants’ opinions about the application, a questionnaire was designed. The objective was to know the impact of the app on their behavior. There was also a question referring to the intake of Metabolaid^®^. The questions included were: (1) how much do you like the app? (value 0–10, with 0 being the lowest, and 10 being the highest); (2) how much is the app helping you to remember to take Metabolaid? (value 0–10, with 0 being the lowest, and 10 being the highest); (3) how much is the app helping you to adopt a healthy lifestyle?; (4) what do you like the most about the app?; and (5) what behaviors have you changed since using the app?

### 2.7. Statistical Analysis

The statistical analysis was performed using the JAMOVI statistics program (Sidney, Australia). Descriptive statistics (mean ± standard deviation) and inference analysis were calculated using the Shapiro–Wilk test to establish that the distribution was normal. Afterward, independent t-tests were performed to compare the baseline values between groups. An analysis of co-variance (ANCOVA) (linear model; time (twice) × groups (MTB-MTBAPP)) was used to analyze the effects of the intervention on the results.

The covariability used was fat mass for blood pressure, since a previous analysis demonstrated that individuals with poor eating habits were those that did not improve, or improved to a lesser degree, their fat mass. The size of the effect Eta squared (η^2^) was used for the interaction time × group. An η^2^ ≥ 0.01 indicates a small effect, ≥0.059 indicates a medium effect, and ≥0.0138 is a large effect. For the variables that presented a significant effect, post hoc Bonferroni tests were performed. The level of significance was established at ≤0.05.

## 3. Results

The objective of the study was to evaluate if the use of a mobile app specifically designed to be used together with a dietary supplement for weight loss and lower blood pressure can have a significant impact on an individual’s health. A total of 37 subjects participated in the research, divided into two groups: MTB (*n* = 18) and MTBAPP (*n* = 19). The baseline characteristics are presented in Table 1. There were no significant differences between groups at baseline.

### 3.1. Dietary Assessment

Diet intake was inputted by the subjects during the first and last weeks of the study (Table 2). No significant differences were observed between MTB and MTBAPP groups regarding the nutritional value of their diets during the beginning of the study. The only difference observed was a significant reduction in protein intake in the MTB group at the end of the study (*p* = 0.010), which was not observed in the MTBAPP group. All other parameters remained unaltered at the end of the study. Regarding diet quality, the MTB subjects reported no changes, during the follow-ups, they commented with the nutritionist about their sensations and indicated that they had noticed a decrease in their hunger. In the MTBAPP group, both in the dietary records and in the app perception questionnaires, they generally reported a higher consumption of water, a higher consumption of vegetables and fruits, together with a decrease in the consumption of processed foods, such as industrial pastries at breakfast and sugary desserts at dinner.

### 3.2. Blood Pressure

**Figure 3:**
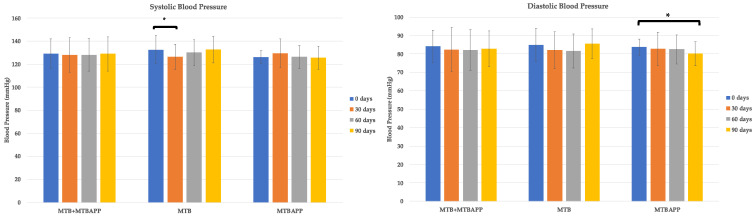
Blood pressure analysis MTB vs. MTBAPP. * *p* < 0.05 by intragroup analysis SBP = systolic blood pressure, and DBP = diastolic blood pressure.

The results of the blood pressure analysis were compared taking into consideration the sum of both study groups (as they both took the same dietary supplement), as well as each group independently. When analyzing the two groups independently, it was revealed that the MTB group significantly reduced their systolic blood pressure (SBP) after 30 days of intake compared with baseline values, however, at days 60 and 90 their levels increased back to initial levels (Figure 3). As for the MTBAPP group, diastolic blood pressure (DBP) decreased throughout the study, being statistically significant after 90 days of intake. A tendency to decrease SBP throughout the study was also observed but did not reach statistical significance. In the MTB group, a 1% increase in DBP was detected at the end of the study, whereas a 4.27% decrease was observed in the MTBAPP group. The analysis of the pooled data, MTB and MTBAPP, did not reveal significant improvement in either SBP or DBP, although a tendency to decrease was detected. Therefore, it can be concluded that the intake of Metabolaid^®^ helps to lower blood pressure, however, in subjects who used the application with nutritional recommendations the reduction in blood pressure was greater, particularly diastolic blood pressure, after 90 days of intake.

### 3.3. Body Weight, Bioelectric Impedance, and Anthropometric Measurements

**Figure 4:**
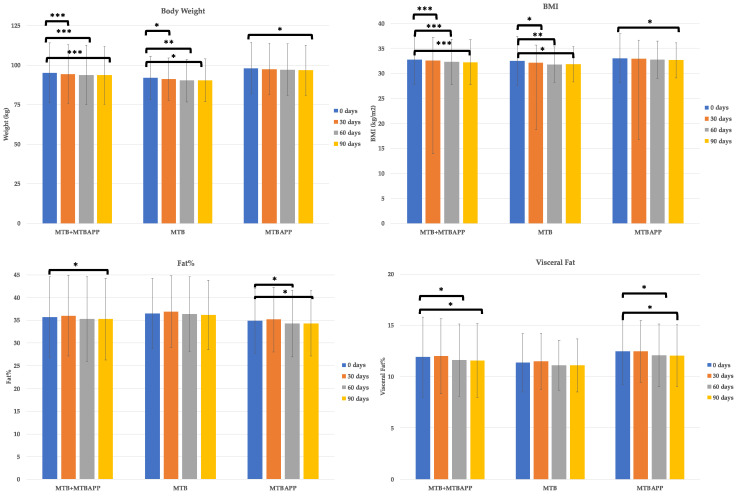
Body weight and body composition data. * *p* < 0.05, ** *p* < 0.01, and *** *p* < 0.005 by intragroup analysis.

Analyzing body weight, BMI, fat mass, and visceral fat, it was observed that both study groups decreased their body weight and subsequently BMI. Regarding body weight and BMI, the combination of the two groups revealed significant improvement starting at the first month, which was also reflected in the MTB group (Figure 4). The MTBAPP group revealed a tendency to decrease starting in the first month, but it was significant after 90 days of dietary intake.

As for body fat, the combination of both study groups revealed a significant decrease in fat percentage and visceral fat, starting at 2 months of treatment. When assessing each study group independently, a significant decrease in fat percentage was detected in the MTBAPP group starting at the second month, while the MTB group did not reveal any significant change. A similar behavior was observed for visceral fat. Therefore, it can be concluded that the ingredient helps reduce body weight and fat mass, particularly visceral fat, and that the app contributed to a greater extent in reducing body fat.

**Figure 5:**
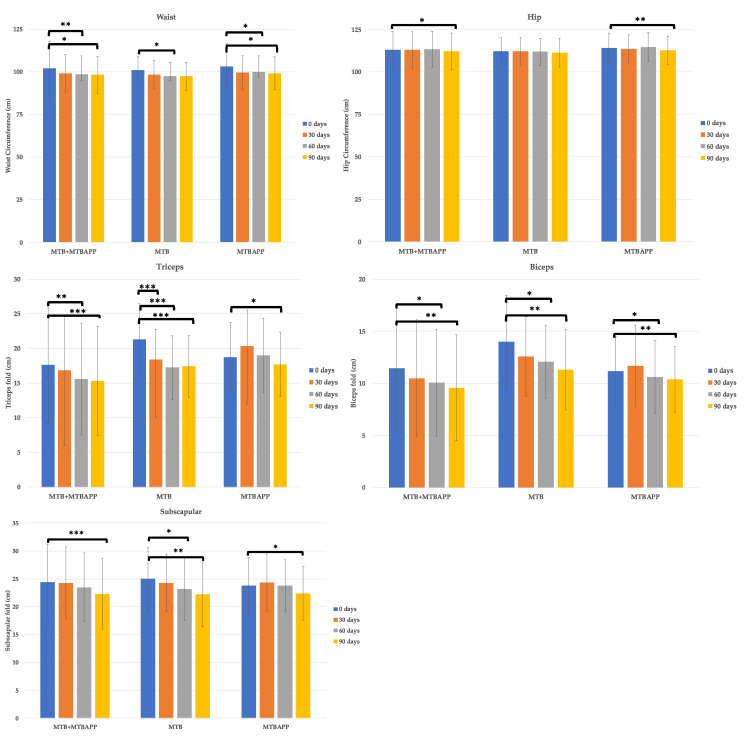
Anthropometric measurements. * *p* < 0.05, ** *p* < 0.01, and *** *p* < 0.005 by intragroup analysis.

Regarding anthropometric measurements, significant differences were observed in both groups in abdominal measurements and skin folds (Figure 5). Waist, hip, triceps, biceps, and subscapular measurements were all significantly reduced in the pooled data. Regarding waist circumference, this parameter was reduced in the pooled data and individual groups starting at 60 days of treatment. In the MTB group, significance was lost at 90 days, while the pooled data and MTBAPP group remained significant at this time point. Regarding hip circumference, significant differences were observed in the pooled data as well as in MTBAPP group at 90 days.

As for the skin folds, the triceps fold was significantly decreased in the pooled data and in both groups individually, being significant from the first month in the MTB group and in month 3 in the MTBAPP group. In the biceps skinfold, a similar effect was observed in the pooled data and in both groups starting at 60 days. Finally, in the subscapular skin fold, significant differences were observed in the pooled data and MTBAPP group at the third month, while the MTB group revealed significant differences starting at the second month.

Therefore, regarding anthropometric measurements, the pooled data and MTBAPP group revealed significant reductions in waist and hip circumferences, while the triceps, biceps, and subscapular folds were significantly decreased in both study groups and the pooled data.

### 3.4. Spirometry, Triglycerides, and One-Mile Walk

No significant differences were observed in any of these tests, neither comparing initial results from final, nor between groups (Table 3).

### 3.5. Physical Activity Data (Fitbit Trackers)

**Figure 6:**
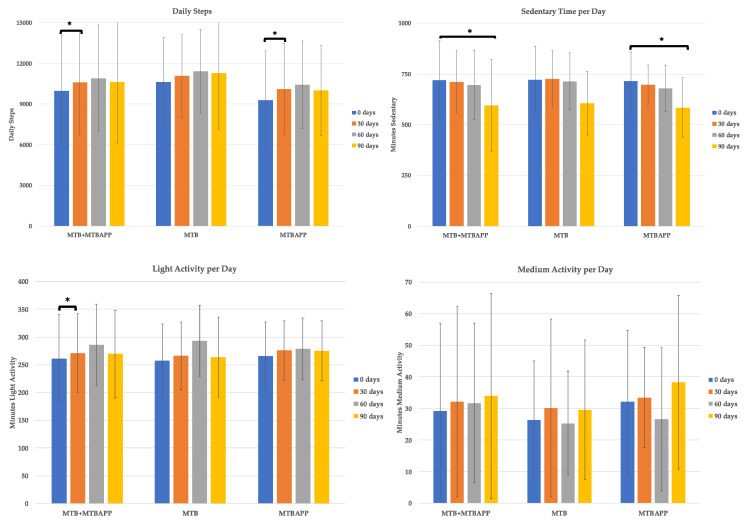

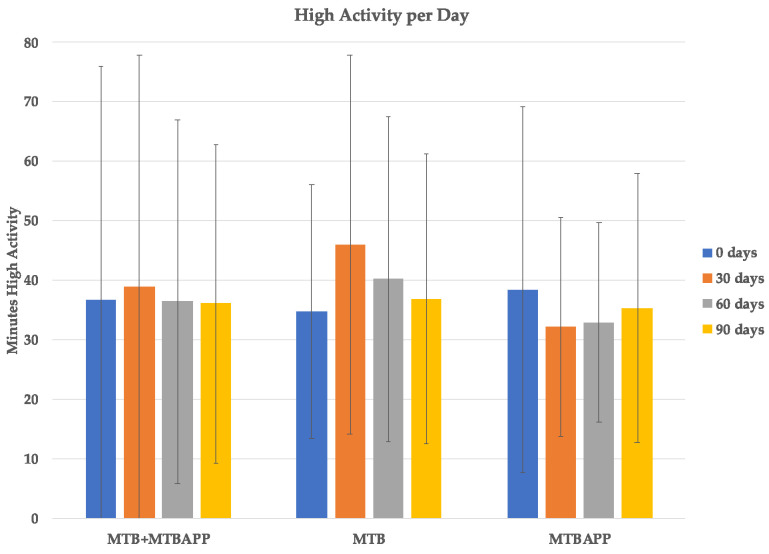
Physical activity data. * *p* < 0.05 by intragroup analysis.

The level of physical activity was measured using the data collected from the Fitbit trackers. In the case of daily steps, a tendency to increase was reported in both study groups, being significant in the MTBAPP group at 30 days, which coincided with the pooled data (Figure 6). However, this was not sustained in time, and significance was lost at months 2 and 3 of the study. As for sedentary time, a significant reduction was detected in the pooled data and MTBAPP group at 90 days of treatment. As for light, medium, and high intensity activity, only light activity was detected to significantly increase, and only in the case of the pooled data at 30 days.

Therefore, the MTBAPP group presented more activity in terms of steps and sedentary time, although a general trend of increase was detected in all groups.

### 3.6. Subject Opinion on App

**Figure 7:**
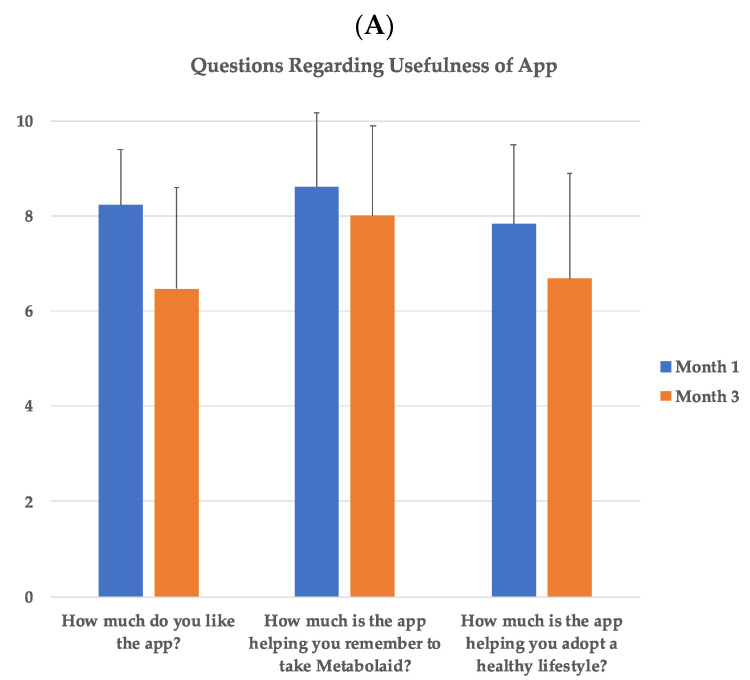

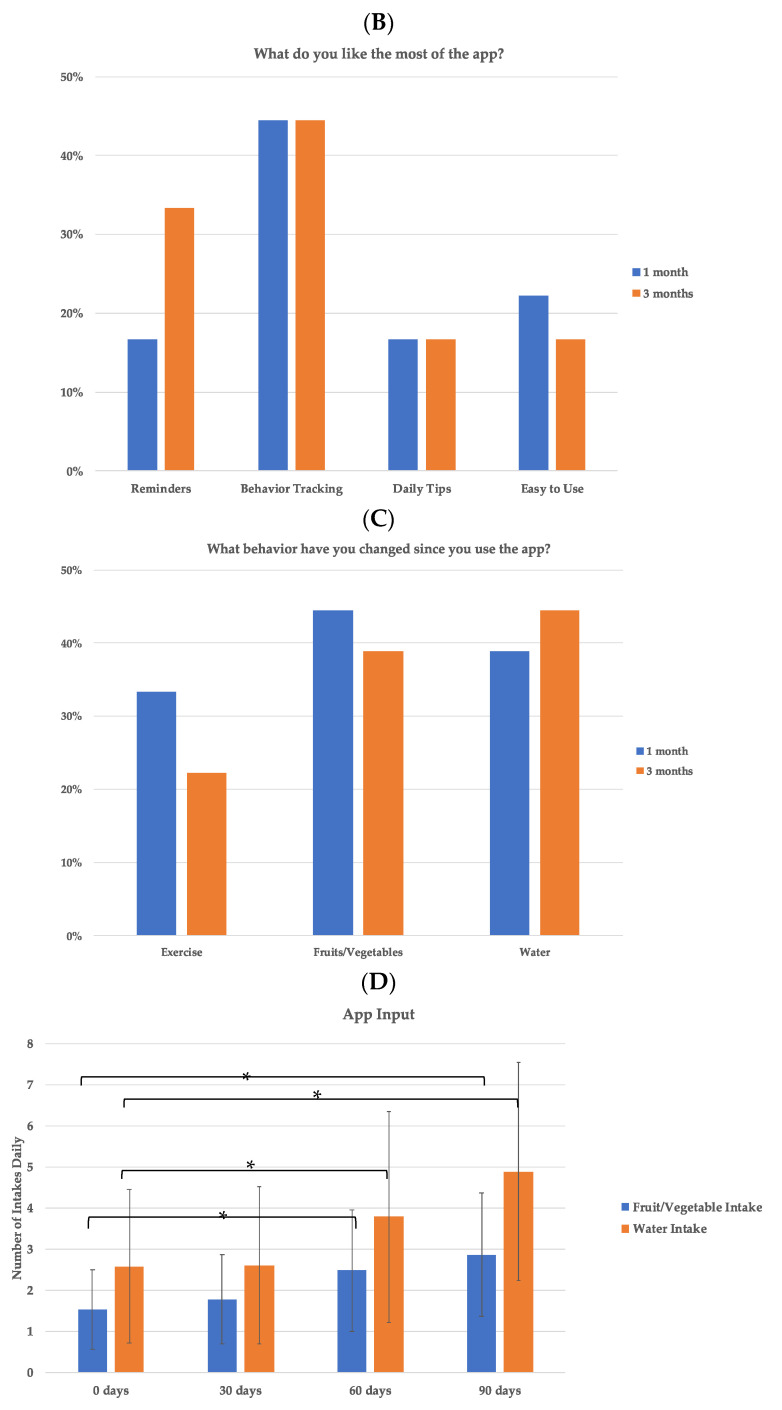
Results of the questionnaire regarding app usage in MTBApp group. (**A**) Results of questions regarding the usefulness of the app, based on a score of 0 (worse) to 10 (best score). (**B**) Percentage of users reporting what aspect did they like the most of the app. (**C**) Percentage of users reporting the behavior that they felt improved the most when using the app. (**D**) Input of the number of fruits/vegetables and water intake during the study. * *p* < 0.001.

Regarding the questionnaire carried out by the subjects in the MTBAPP group to find out their opinion on the use of the app, a positive response was observed (Figure 7A–D) in terms of liking and usefulness of the app. They considered it as a useful tool to help adopt a healthy lifestyle, with a score of 8 out of 10 on average on these questions. This value remained unchanged after the 3 months of the study, although a downward trend was observed.

When asked what was the feature that they liked the most, behavior tracking was the most commonly named feature both after 1 month and 3 months of the study. The daily reminders, daily tips, and ease of use were evenly mentioned as their favorite features, where the reminders increased in importance after 3 months compared with the first month.

Finally, when asked what lifestyle behaviors they had changed since using the app, fruit/vegetable and water intake were the most commonly mentioned behaviors, followed by exercise. This was corroborated by the manual input of intake in the app, where these values increased progressively throughout the study, being significant starting at day 60 of treatment.

## 4. Discussion

The current study reveals that the use of a mobile application, as part of a weight management strategy that includes lifestyle recommendations and daily tips on how to adopt them to the user´s everyday life, can contribute to an increase in physical activity and ultimately provide significantly better results compared with non-app users following the same intervention.

A recent meta-analysis [25] of 41 studies, involving more than 6300 participants, showed that app-based mobile interventions can be effective in changing nutritional behaviors and their main nutrition-related health outcomes in a wide range of study settings. The present research confirms these findings. Both study groups managed to lose weight and body fat. Regarding fat, a significant decrease was observed in fat% and visceral fat only in the MTBAPP group, as well as in the pooled data. Therefore, it appears, and as previously observed in mouse studies [26], that the Metabolaid ingredient reduces both body weight and body fat. In addition, this effect is enhanced when the intake of the nutraceutical is complemented by a specific app. This loss of fat mass was corroborated by the lower waist and hip circumference, suggesting that the use of the app had an influence on the improvement of these variables. However, significant blood pressure drop was detected in the MTBAPP only in the long term, particularly in the diastolic blood pressure. Previous studies have demonstrated that the dietary ingredient Metabolaid can contribute to reducing blood pressure in pre-hypertensive individuals and body weight [15,16,27,28]. This is at least due in part to the antioxidant and anti-inflammatory properties of the lemon verbena [29], which has been shown to reduce lipid accumulation both in the fat tissue as well as in the liver [30]. Furthermore, the hibiscus extract has been shown to contribute to lowering cholesterol and triglycerides, fat accumulation, as well as lower blood pressure [16,31,32]. In the present investigation, it seems that the intake of the nutraceutical has not been sufficient, possibly due to the lower number of subjects compared with previous studies, although a tendency to decrease was detected. On the other hand, the combination of the nutraceutical and app did present significant results, possibly due to the app promoting habits that are related to better cardiovascular health (proper nutrition, physical exercise, adequate water intake, among others).

Regarding physical activity, the MTBAPP group significantly increased their daily steps during the first month of the study with an over 14% increase at the first and second month (from approx. 9000 daily steps to 10,500 steps), but dropping in the third month (yet still 9.5% higher than baseline). This was also reflected in less sedentary minutes in the app users. The MTBAPP group overall gave positive feedback regarding the usefulness of the app throughout the whole study duration, with the behavior tracking considered as the best-liked feature. In the research conducted by Liliana Laranjo et al. in 2020 [33], they also counted steps after the use of a mobile app, using Fitbit devices. The results partially coincide (in the present research the participants were overweight), as they observed that the normal weight group increased their daily step count during the duration of the study by an average of 2292 steps, however, the overweight and obese group did not show statistically significant changes. However, research analyzing [34] step counts of users subscribed to the Sweatcoin app using motivational incentives showed that overweight individuals appear to benefit most from incentive-based physical activity programs due to their low intrinsic motivation but minimal physical barriers to increasing activity levels.

The current study included a mobile app specifically designed to be used alongside a dietary supplement that includes the ingredient Metabolaid^®^. In addition to the above, Metabolaid^®^ also helps to reduce appetite levels, thus helping consumers to control their hunger [15,35]. Information about the ingredient, its properties, benefits, and expected results was included in the application for users to access.

Regarding lifestyle modifications, the app users revealed that introducing more fruits and vegetables in their diet, and increasing water intake, were the most frequently adopted changes, which was reflected in the increased intake observed in the app. Pooling together the data from both groups revealed that Metabolaid significantly reduced systolic blood pressure, while diastolic blood pressure presented a noticeable, yet not quite significant decrease. The lack of significance in the MTB group could be due to the lower number of participants compared with the other studies [15,16,27,36]. Moreover, the average blood pressure values in the previous studies were higher compared with the current study. Finally, a single-arm measurement was performed in the current study, whereas previous studies used continuous blood pressure measurements for 24 h. These reasons could be behind the differences observed with previous studies.

As previously mentioned, treatment adherence is a matter of grave concern, particularly in chronic conditions [37,38]. The same occurs with dietary supplements, where a high dropout rate is common. Many times, it is a matter of forgetfulness, therefore, one of the features introduced into the app is the inclusion of a daily reminder to take the product at the most adequate moment for the user. In this study, digital technology is used as a facilitator, but not as part of the treatment. For this reason, the reminder is added as an optional feature that the user can decide to not use (although the app recommends activating the reminder until the product intake becomes a habit) and can have it activated in the days of the week and moments that work best for the user.

Joshua H. West et al. [39] observed that diet and nutrition-related apps that focus on improving motivation, desire, self-efficacy, attitudes, knowledge, and goal setting can be particularly useful. It is for this reason that motivation management was taken into account when designing the application. As occurs with any new behavior change, maintaining user motivation for a prolonged amount of time can be a difficult task. At first, user motivation is high, however, as time goes by, it drops successively until the user either drops out or a new motivational trigger appears. In this case, several strategies were implemented in the app, based on behavior change techniques [40,41]. First, a weekly challenge was implemented in the app, where each week the user was recommended to focus on a small behavior change (drink more glasses of water, add more fruits/vegetables, do more exercise). This makes the app more dynamic, adding a new goal every week, and therefore less monotonous. Moreover, a point score system was included as a method to reward the efforts of the user. Each time the user successfully reached a daily goal (take eight glasses of water, five pieces of fruits/vegetables, 30 min of physical activity, take the dietary supplement), the user was rewarded with a number of points, which were added up to a total score. In addition, a ranking of all the users was included, so that they can visualize how they compare with other users, which is a proven method to increase motivation. This provided the user with information on how their level of compliance throughout time was, and could even motivate them to improve their behavior, and their score, in successive days and weeks.

Lastly, educational material was added to the app. It has been observed that providing nutritional education with the objective of promoting lifestyle changes, through digital devices, has positive effects in both the short and long term in the treatment of chronic diseases such as diabetes [42], obesity, or hypertension [43]. This is why it was provided in the form of text pages on the corresponding screen (educational material regarding exercise in the physical activity screen, etc.), providing information on the importance of the behavior habit. In addition, a pop-up notification was sent daily with a behavioral tip on how to introduce those new habits.

Overall, the combination of these features in the app provides a more holistic approach to improve user lifestyle behavior. It reveals to the user that searching to lose weight and lower their blood pressure requires more than just taking a dietary supplement, and that lifestyle changes are necessary. This observation was demonstrated by the users’ questionnaire responses, where they reported the importance of using the app to reach their goals, as well as what features they found most useful. Taken together, having closer and more direct contact with subjects by offering them constant information on how to improve their diet, even in the form of a mobile application, can increase the success rate of adopting lifestyle behaviors.

As a strength, it is possible to include the use of the app as such, since it has been seen to improve the health of the participants, improving their autonomy in the control of their eating habits and physical activity. All participants lived in the same geographical area, so the objectives, design, and environment were similar. There are some limitations to this study, firstly, the sample size and the short period of time the intervention was conducted (3 months). Future research should be aimed at improving the understanding of the time course of the effects of the intervention, both of Metabolaid intake and app use. In particular, a greater understanding is required for the timing of the peak effect size, the timing of the decline in user engagement, de-motivation, and the factors underpinning these phenomena. This may involve future studies with longer follow-up periods and with outcome measures taken at more regular and frequent time points. The relatively short-term nature of the positive effects suggests that additional efforts are required to design app features that help maintain user engagement with the app over time, for example, perhaps through modules, unlockable content, and rewards.

## 5. Conclusions

In conclusion, the study presented demonstrates that the use of a mobile application specifically designed to help users lose weight and adopt a healthy lifestyle can have a more significant impact on the subject´s health than non-app users following the same intervention. Specifically, where both study groups lost body weight (*p* < 0.001) and localized fat (*p* < 0.005), the app users managed to lower their blood pressure (*p* < 0.005) and increase their level of physical activity (more steps, less sedentary minutes). This provides further proof that mobile apps can be a valuable tool to help users adopt healthy lifestyle habits and improve their health.

## Figures and Tables

**Table 1:** Demographics of MTB and MTBAPP subjects.

	MTB (*n* = 18)	MTBAPP (*n* = 19)	*p* Value
	Mean ± SD	Mean ± SD	
Age (years)	46.1 ± 10.4	45.3 ± 6.40	0.858
Systolic BP (mmHg)	132 ± 16.3	126 ± 7.11	0.117
Diastolic BP (mmHg)	84.9 ± 11.7	83.8 ± 4.69	0.695
Height (cm)	167 ± 10.1	172 ± 8.47	0.322
Weight (kg)	90.9 ± 17.0	99.1 ± 21.6	0.359
BMI (kg/m^2^)	32.7 ± 4.75	33.2 ± 5.52	0.841
Fat %	37.0 ± 9.30	34.8 ± 8.53	0.606
Visceral Fat %	11.4 ± 3.70	12.9 ± 4.59	0.399
Waist (cm)	101 ± 10.5	105 ± 22.3	0.706
Hip (cm)	112 ± 10.7	115 ± 11.7	0.585
Abd. perimeter (cm)	108 ± 12.0	113 ± 16.4	0.577
Triceps folds (mm)	21.9 ± 6.30	19.0 ± 6.76	0.251
Bicep folds (mm)	14.4 ± 5.36	10.9 ± 3.98	0.076
Subscapular folds (mm)	24.6 ± 7.18	22.1 ± 6.06	0.573
PEF (L/m)	373 ± 150	520 ± 168	0.510
FEV1 spirometry (L)	3.02 ± 0.954	3.54 ± 0.716	0.198
Triglycerides (mg/dL)	138 ± 86.8	156 ± 138	0.674
Time walk 1 mile (min)	16.8 ± 2.33	16.8 ± 3.22	0.526
Heart rate post-walk (bpm)	121 ± 11.6	131 ± 14.2	0.515
VO_2_ max (ml/kg/min)	28.0 ± 12.6	26.4 ± 11.0	0.909

Data are presented as mean ± standard deviation. BP = blood pressure; cm = centimeters; mm = millimeters; FEV1 = forced expiratory volume in the first second; PEF = peak expiratory flow; kg = kilograms; % = percentage; L = liters; mg = milligrams; bpm = beats per minute; and mL = milliliters.

**Table 2:** Diet assessment of MTB and MTBAPP.

	0 Days	90 Days	0 Days	90 Days	Effect Time × Group		
	Mean ± SD	Mean ± SD	Mean ± SD	Mean ± SD	F	*p*	η^2^
Kcal	1806 ± 511	1722 ± 483	1796 ± 431	1774 ± 337	0.413	0.525	0.012
Carb (g)	175 ± 59.2	173 ± 57.2	166 ± 45.2	168 ± 41.9	0.062	0.804	0.002
Prot (g)	102 ± 31.9	78.6 ± 27.5	94.2 ± 19.5	92.6 ± 14.7	5.580	0.024 *	0.138
Lip (g)	75.7 ± 28.7	73.0 ± 23.1	78.7 ± 22.8	76.3 ± 20.9	0.001	0.965	0.000
Vit. B12 (mg)	4.51 ± 2.48	5.22 ± 4.16	6.80 ± 3.32	7.10 ± 6.43	0.047	0.829	0.001
Na (mg)	1783 ± 1403	2019 ± 1598	2604 ± 1323	2345 ± 1488	125.4	0.270	0.035
Ca (mg)	755 ± 330	841 ± 325	697 ± 286	759 ± 311	0.030	0.862	0.001
Fe (mg)	2.26 ± 1.18	1.92 ± 0.836	2.42 ± 0.423	2.16 ± 0.412	0.037	0.847	0.001
Vit. C (mg)	121 ± 81.6	127 ± 82.3	158 ± 87.3	158 ± 75.0	0.047	0.829	0.001
Vit. D (µg)	46.1 ± 63.9	10.6 ± 17.3	4.65 ± 3.92	54.5 ± 95.6	9.359	0.104	0.211
Chol. (mg)	340 ± 274	315 ± 190	322 ± 111	351 ± 125	0.451	0.506	0.013

Data are presented as mean ± standard deviation. Kcal = kilocalories; carb = carbohydrates; prot = proteins; lip = lipids; vit = vitamin; Na = sodium; Ca = Calcium; Fe = Iron; chol = cholesterol; g = grams; mg = milligrams; and µg = micrograms. Mean differences are considered significant when *p* < 0.05, and * intra-group differences MTB.

**Table 3:** Spirometry, triglycerides, and time to walk one mile data at the beginning and end of the study.

	MTB		MTBAPP	
	0 Days	90 Days	0 Days	90 Days
	Mean ± SD	Mean ± SD	Mean ± SD	Mean ± SD
Spirometry PEF (L/min)	378.94 ± 147.60	398.78 ± 131.36	507.84 ± 157.48	496.63 ± 160.19
Spirometry FEV1 (L)	3.08 ± 0.95	3.18 ± 0.78	3.44 ± 0.70	3.45 ± 0.63
Triglycerides (mg/dL)	169.46 ± 76.20	204.00 ± 84.37	189.32 ± 121.64	163.42 ± 34.21
Time to walk 1 mile (min.)	17.19 ± 2.81	16.53 ± 2.46	16.56 ± 3.10	15.69 ± 2.37

SD = standard deviation; L = liters; m = minutes; mg = milligrams; and dL = deciliters.

## Data Availability

The data presented in this study are available on request from the corresponding author. The data are not publicly available due to personal health information.
